# BASILIScan: a tool for high-throughput analysis of intrinsic disorder patterns in homologous proteins

**DOI:** 10.1186/s12864-018-5322-5

**Published:** 2018-12-11

**Authors:** Michal Barski

**Affiliations:** Section of Virology, Department of Medicine, St Mary’s Hospital, Imperial College London, London, W2 1PG UK

## Abstract

**Background:**

Intrinsic structural disorder is a common property of many proteins, especially in eukaryotic and virus proteomes. The tendency of some proteins or protein regions to exist in a disordered state usually precludes their structural characterisation and renders them especially difficult for experimental handling after recombinant expression.

**Results:**

A new intuitive, publicly-available computational resource, called BASILIScan, is presented here. It provides a BLAST-based search for close homologues of the protein of interest, integrated with a simultaneous prediction of intrinsic disorder together with a robust data viewer and interpreter. This allows for a quick, high-throughput screening, scoring and selection of closely-related yet highly structured homologues of the protein of interest. Comparative parallel analysis of the conservation of extended regions of disorder in multiple sequences is also offered. The use of BASILIScan and its capacity for yielding biologically applicable predictions is demonstrated. Using a high-throughput BASILIScan screen it is also shown that a large proportion of the human proteome displays homologous sequences of superior intrinsic structural order in many related species.

**Conclusion:**

Through the swift identification of intrinsically stable homologues and poorly conserved disordered regions by the BASILIScan software, the chances of successful recombinant protein expression and compatibility with downstream applications such as crystallisation can be greatly increased.

**Electronic supplementary material:**

The online version of this article (10.1186/s12864-018-5322-5) contains supplementary material, which is available to authorized users.

## Background

The incidence of intrinsic disorder – defined as the lack of a fixed three-dimensional conformation of a protein - has become increasingly appreciated and examined in recent years. Many such proteins have been characterised, and their disorder, as well as order-disorder transitions upon ligand and partner binding demonstrated with a range of biophysical techniques [1]. It is now widely acknowledged that protein disorder is a remarkably common phenomenon – especially in complex organisms and viruses. An estimated 30–40% of the human proteome is disordered to a significant degree [2]. This has far-reaching consequences for the structural characterisation and experimental handling of many proteins from the human and other disorder-enriched proteomes.

Depending on the extent and location of flexibility, the intrinsically-disordered protein (IDP) can exhibit a number of different behaviours. Although the highly-charged nature of disordered regions may confer high solubility [3], presence of proteases in IDP preparations often causes severe proteolytic degradation in the case of fully-disordered IDPs or digestion of long connecting loops within and between otherwise structured domains [4, 5]. Ultimately, biochemical and biophysical characterisation of such proteins is usually difficult because of challenging protein expression and purification and lack of sample homogeneity. Crystallisation of IDPs for X-ray crystallography is only feasible with the flexible regions removed, bound to a co-factor, or entropically-stabilised otherwise. Multidimensional nuclear magnetic resonance (NMR) spectroscopy remains the only high-resolution biophysical technique for studying some disordered protein systems, although the sample has to meet stringent compatibility standards (small and globular proteins, homogeneity, stability under low ionic strength for long periods of time) [6].

Since IDPs display characteristic patterns of amino acid content and distribution, the presence of disordered regions can be predicted from their primary sequence with high confidence. Many predictive algorithms have been devised and used successfully to predict the probability of residues in a given protein sequence to exist in an ordered or disordered state [7–11]. Such predictions have been repeatedly confirmed experimentally with multi-dimensional NMR [12, 13]. It is currently becoming common practice to take the intrinsic disorder predictions into account while designing a protein construct for recombinant expression. The N- or C-terminal disordered tails/domains can be truncated (for example: [14, 15]), while long loops connecting neighbouring domains or elements of secondary structure can be shortened to aid conformational stability [16]. Unfortunately, the thin line between limiting disorder and affecting the function and correct folding of the protein can easily be crossed and the experimental trial-and-error process often takes long before an improved construct is found.

The software presented here, called BASILIScan, offers an alternative approach to streamline and simplify the construct design process. The core mechanism relies on a BLASTP search with the user’s amino acid sequence linked with simultaneous intrinsic disorder prediction of all hits. A specialised scoring system, called the FLEX score, is then employed to identify closest homologues exhibiting lowest disorder content. A hit possessing a FLEX score superior to the submitted protein is very likely to also show improved in vitro behaviour. Analytical features of BASILIScan, such as multiple sequence disorder overlay and alignment, allow for analysis of disorder conservation patterns between selected homologous hits in parallel. In addition to identifying a more suitable homologue, such analysis can guide rational construct design by exposing intrinsically-disordered regions of low or limited conservation which could be truncated from the expression construct. The adjustability of numerous search and analysis parameters provides compatibility with protein sequences of a wide range of intrinsic disorder, length and inter-species sequence variation.

## Implementation

**Fig. 1:**
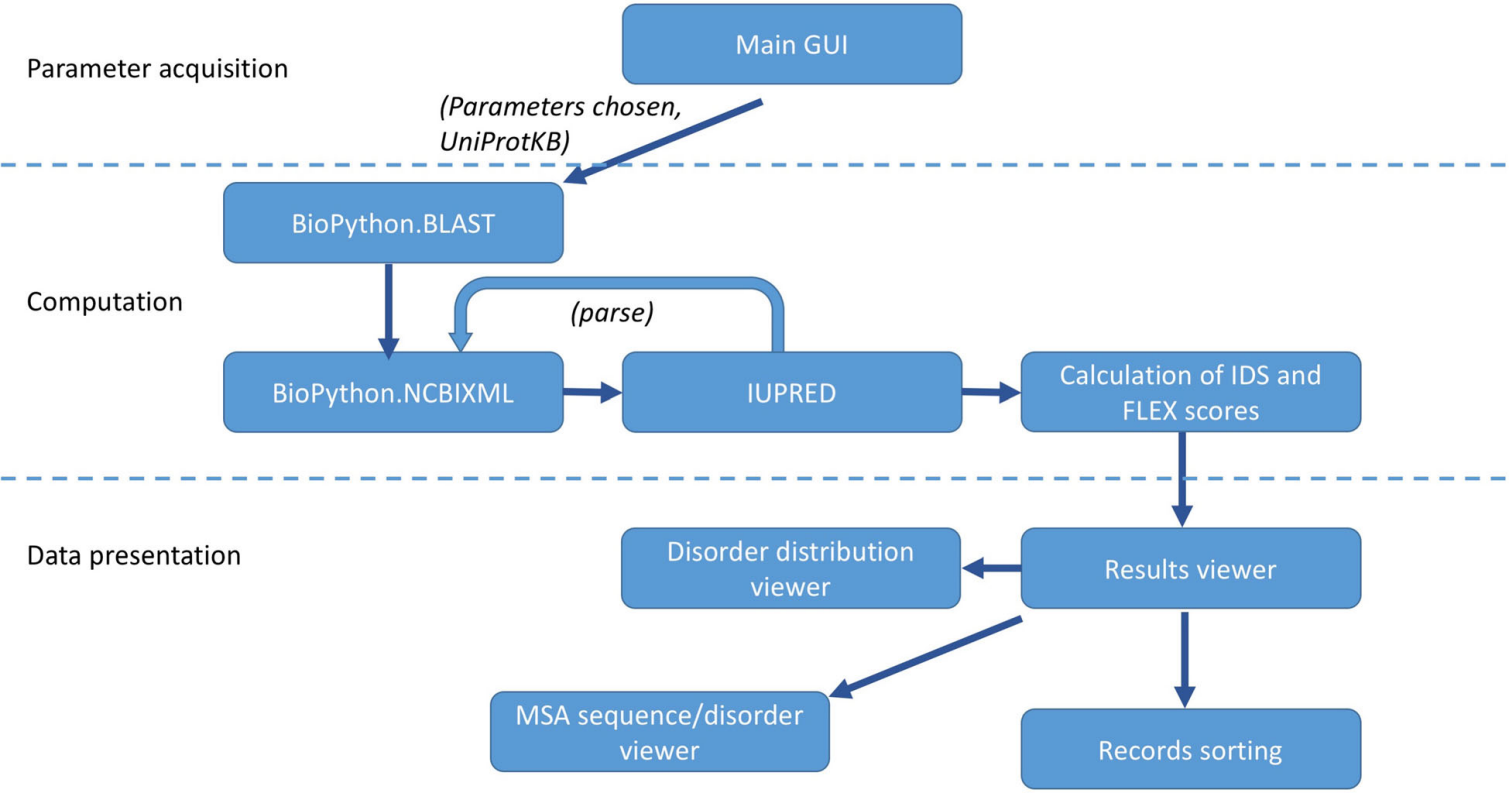
The component architecture of BASILIScan, classified into the three stages of: parameter acquisition, computation and data presentation

The main framework of the BASILIScan software is centred upon the connection of the modules outlined in Fig. 1, written in Python 2.7. Where possible, Biopython (ver. 1.67) [17] libraries or their derivatives are used. In order to promote the multi-disciplinary use of BASILIScan, a graphical user interface (GUI) has been created in Tkinter ver. 8.5.9 for Python.

### Sequence-based similarity search

After the plain unformatted amino acid sequence is provided, a BLASTP [18] search is conducted against the selected sequence database (UniprotKB/Swissprot being the default and recommended one [19]) and the results displayed according to the filters set by the user – such as an expect value (E-value) threshold or a maximum number of hits. The Bio.Blast functionality of Biopython is used to run on-line BLAST. The .xml output file is parsed with the NCBIXML function in Biopython.

Users may perform searches on custom, remote, FASTA-formatted libraries instead, by selecting the library file through the “Advanced properties/Select database” option. Remote search on the selected library is then performed by BLAST+ ver 2.7.1, including conversion of the library by makeblastdb. Sequence identifiers used in remote libraries should be Uniprot-derived in order for all BASILIScan modules to work correctly.

### Handling of viral polyproteins

In the case of the resulting sequences constituting a viral polyprotein, BASILIScan will perform virtual processing of the polyprotein and will apply all its analysis and metrics tools to the appropriate fragment only. This functionality is only offered when UniprotKB/Swissprot database is selected for search, due to lack of proteolytic processing information in non-manually curated databases. This option can also be disabled in “Edit/Advanced preferences”.

### Prediction of intrinsic disorder

For BLAST hits fulfilling the criteria set by the user, intrinsic disorder is calculated by the IUPRED algorithm [11], which has been used extensively in other publications for predicting intrinsic disorder in silico [20–23]. Two alternative IUPRED modes are available: “long disorder” or “short disorder”. For most applications, the “long disorder” mode is suggested. The intrinsic disorder score (IDS) of each entry is then calculated in the following way:

$$ IDS=\frac{\sum_{r=1}^l{\gamma}_s}{l}\times 100\%\kern0.5em where\ {\gamma}_s=1\  if\ s>0.5\ \left( or\ user\ selection\right); otherwise\ {\gamma}_s=0 $$

where *l* is the length of the protein and *s* is the residue’s IUPRED score.

### FLEX score computation

Since identification of a homologue with superior intrinsic disorder properties requires scoring at least two parameters simultaneously, the hybrid FLEX score has been implemented. The FLEX score incorporates a weighted average of the intrinsic order and a hyperbolic transform of the E-value parameter in the following fashion:

$$ FLEX\ score=\left(\left(1-\eta \right)\left(1-{0.99}^{{\left(-\log \left(E- value\right)\right)}^2}\right)+\eta \left(1- IDS\right)\right) $$

The weight is determined by the FLEX coefficient (*η*), which is set by the user before the homology search is run. Allowed values are between 0 and 1 and will shift the contribution ratio of intrinsic structural order (*1 - IDS*) to E-value transform. The hyperbolic transform of the E-value is meant to converge the extreme low-end E-value range while resolving the high-end and middle ranges. Consequently, the function of the logarithm of the E-value is sigmoidal, and bound from 0 to 1. It is characterised by a near-linear relationship between arguments corresponding to E-values of 10^− 3^ and 10^− 14^, while either tail approaches 0 or 1, respectively (Additional file 1: Figure S1).

### Visualisation of results

Results of a sequence query are presented in a table, for each hit showing the UniProt identifier, the GeneID, the expect value (E), sequence identity, similarity, the IDS score and the FLEX score. By default, the result hits are sorted from the lowest to the highest E-value. Sorting priority can be adjusted at any time from the main menu. The right-hand-side menu allows for more in-depth analysis of results. The ‘View’ option acquires the most important parameters of the selected item from the UniProt repository, displaying information such as sequence length, molecular mass and organism taxonomy.

The “Details” button draws a detailed trace of intrinsic disorder of the selected record within an interactive two-coordinate environment, implemented with Matplotlib. The environment allows for enlargement of selected parts of the trace, as well as for its translation. The option of exporting the graph as an image file is also provided. Traces can be overlayed on top of each other and therefore the intrinsic disorder can be explicitly compared between multiple protein records simultaneously.

Importantly, if the “enable disorder trace alignment” setting is switched on, the multiple disorder traces for the selected protein records will be automatically aligned on the axis, according to a multiple sequence alignment conducted in CLUSTALW [24]. Default CLUSTALW parameters can be adjusted in Edit/Advanced properties. Any gaps inserted through the alignment algorithm will be visible in the aligned traces as residues with the IUPRED disorder score of 0.0 – an extremely unlikely occurrence for a protein residue otherwise.

### Distribution

Windows and OSX binary distributions of BASILIScan were packaged with Py2exe and Py2app, respectively. Packages for both platforms are freely available for academic use under the GNU distribution license and can be downloaded at www.basilisc.com/downloads. Open-source version is also available. Please consult the ReadMe file for further instructions on installation and running of BASILIScan, as well as for the dependencies required to run the open-source version (www.basilisc.com/readme/).

## Results

### Case scenario: Human CDC7 protein kinase

Every BASILIScan job starts with providing a raw amino acid sequence and a job title. Next, the IUPRED mode is selected. Unless one is looking for very short stretches of disorder, or within very short protein sequences, the “long disorder” mode should be selected. The “E-value threshold” indicates the upper limit of the BLAST E-value, above which any hits will be ignored. The number of homology hits found can also be capped. Lastly, the “FLEX score priority coefficient” has to be set. This parameter will not influence which results are shown, but is meant to adjust the sensitivity of the FLEX score by shifting the contribution of homology versus structural order towards the final score. This means, for instance, that when the “priority coefficient” is set to 100% (all the way towards structural order), the FLEX score will only reflect the intrinsic order (*1-IDS*) of a given hit and will ignore the homology score component. The adjustment of the “priority coefficient” is particularly useful in cases where the BASILIScan run yields many hits containing one or more subpopulations clustered around particular values of IDS or E-value. The default priority coefficient value is 50%, in which case both terms will be taken into account equally.

**Fig. 2:**
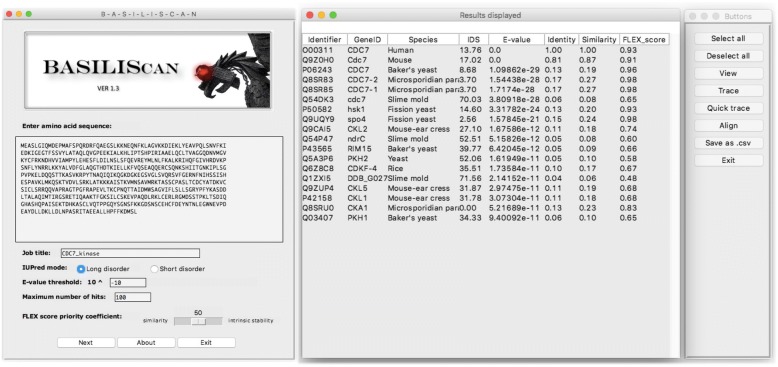
Presentation of search results by the BASILIScan GUI. The left panel provides a choice of basic parameters. Other parameters can be accessed through the “Advanced Properties” menu button. The middle panel shows the most important characteristics and calculated parameters of each result hit. Upon selection of one or more hit identifiers, the right panel buttons will activate different analysis modules

To showcase the functioning of BASILIScan, the human cell division cycle protein kinase 7 (hCDC7, Uniprot identifier: O00311) was chosen as the sequence of interest. The protein is a 63.9 kDa serine/threonine protein kinase, which is an essential S-phase kinase implicated in DNA replication control and cell proliferation [25]. The choice of hCDC7 kinase was dictated by availability of subject literature and structural information, as well as its functional conservation across many species. The 574-amino acid sequence of hCDC7 was submitted to BASILIScan for search in “long disorder” IUPRED mode, with an E-value threshold of 10^− 10^, maximum number of hits of 100 and a FLEX score priority coefficient of 50%. Figure 2 and Additional file 1: Table S1 show, respectively, the way results are displayed by the BASILIScan GUI and a full results dataset from the above search.

**Fig. 3:**
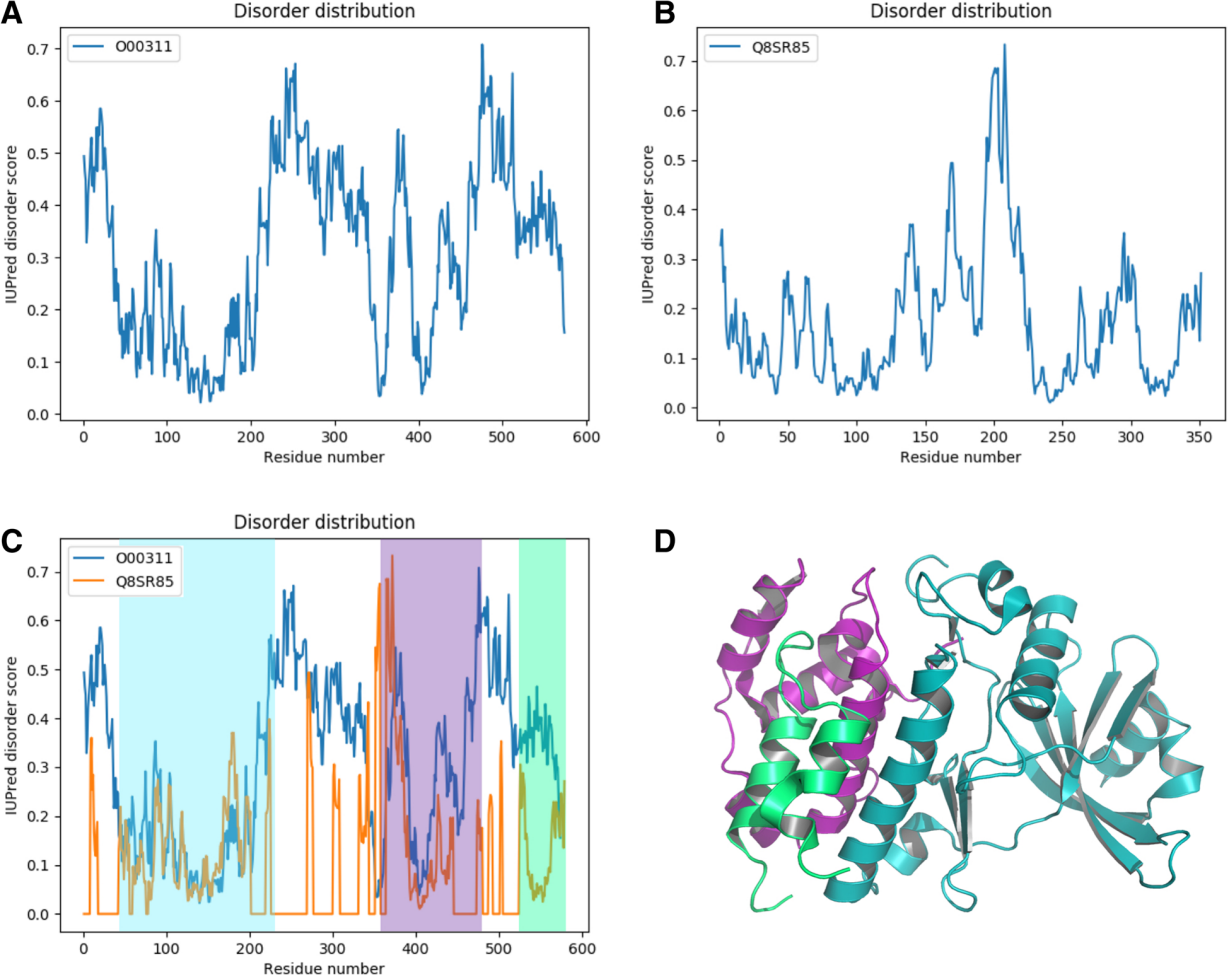
Detailed traces of putative intrinsic disorder drawn by the “Trace” module (**a**-**c**). Extensive regions of disorder are apparent from the disorder trace of human CDC7 kinase (**a**). The disorder trace of the highest-scoring hit in the BASILIScan search, the CDC7 kinase homologue of E. cuniculi, reveals a much more ordered appearance (**b**). Disorder trace alignment module allows for identification of the disordered regions in human CDC7 (blue trace) that are missing in the fungal homologue (orange trace). The resulting structured domains are highlighted in cyan, purple and green (**c**). A deletion construct of human CDC7 kinase composed of the regions highlighted in panel **c** has been successfully crystallised and its structure solved to 2.3 Å resolution (PDB: 4F99) [26] (**d**) Regions of the structure in panel D are coloured corresponding to the highlighting colour in panel **c**

The BASILIScan search yielded 18 hits, including the search query entry which would usually appear as the first row in the results window and always returns the E-value of 0. It is immediately apparent that although many homologues with low E-values were found, their intrinsic disorder varies considerably (from 0 to 72%). Human CDC7 kinase shows an IDS score of about 14%, with many residues oscillating around the 0.5 disorder threshold. This can be seen by using the module “Trace” which graphs the disorder scores for every residue in a given sequence (Fig. 3a). Indeed, lowering the IUPRED score calculation threshold from the default 0.5 to 0.4, leads to a dramatic increase in IDS of hCDC7 up to 35%. The FLEX score, being a weighted average of the E-value transform and the IDS score, should in most cases serve as the easiest measure of identifying suitable hits. The most promising hit returned by this BASILIScan search, possessing the highest FLEX score, is a probable CDC7 kinase homologue from a fungus *Encephalitozoon cuniculi*. In addition, a remote search with BASILIScan against a TrEMBL database encompassing all available vertebrate proteomes has also found numerous evolutionarily-closer homologous sequences with IDS and FLEX scores superior to the search query statistics (Additional file 2).

Overlaying the calculated intrinsic disorder trace of the submitted sequence (O00311) on top of the BASILIScan hit of the highest FLEX score (Q8SR85), clearly shows the conservation of some intrinsic disorder patterns (residues 363–390) and confirms that there are regions of diminished intrinsic disorder (e.g. residues 420–446, 532–574) as well as significant deletions of particularly disordered segments (residues 1–40, 202–331) in the fungal and vertebrate homologues (Fig. 3a-c). The “align” feature can also be used to perform a multiple sequence alignment on multiple selected hits and overlay the calculated intrinsic disorder scores for each residue in the alignment as a heatmap, in order to compare the conservation of disorder between many sequences at once (Additional file 1: Figure S2).

The crystal structure of human CDC7 kinase has been reported [26]. Crystallisation was achieved through identification of disordered regions by limited proteolysis and NMR spectroscopy, with their subsequent deletion. The crystallised protein encompasses the three ordered regions predicted by BASILIScan (highlighted in cyan, purple and green in Fig. 3c-d).

The parametrisation offered by BASILIScan, in particular the “priority coefficient”, allows for a quick assessment of the results, targeted to one’s needs. This is especially useful when many result hits are returned. Resubmitting the above CDC7 kinase query with the E-value threshold lowered to − 5, the maximum set number of 100 hits are obtained (Table 1). Since many homologues with a low E-value are returned, it is easier to assess the results by shifting the priority coefficient towards intrinsic stability (i.e. higher value). In this instance, there will be many more hits with low IDS scores among the top FLEX score results. Changing the priority coefficient in the opposite direction will have an opposite effect.

**Table 1:** Summary of the top 20 BASILIScan results for homologues of human CDC7 kinase (Uniprot/Swissprot identifier O00311) with the priority coefficient set to either 10% or 90%

Priority coefficient = 10%						Priority coefficient = 90%					
Identifier	GeneID	Species	IDS	E-value	FLEX_score	Identifier	GeneID	Species	IDS	E-value	FLEX_score
Q8SR83	CDC7–2	Microsporidian parasite	3.703	1.54E-28	0.9959	Q9UQY9	spo4	Fission yeast	2.564	1.58E-21	0.9756
Q8SR85	CDC7–1	Microsporidian parasite	3.703	1.72E-28	0.9959	Q8SR83	CDC7–2	Microsporidian parasite	3.703	1.54E-28	0.9666
P06243	CDC7	Baker’s yeast	8.678	1.10E-29	0.9911	Q8SR85	CDC7–1	Microsporidian parasite	3.703	1.72E-28	0.9666
O00311	CDC7	Human	13.76	0	0.9862	Q8SRU0	CKA1	Microsporidian parasite	0	5.22E-11	0.9654
Q9UQY9	spo4	Fission yeast	2.564	1.58E-21	0.9858	P40231	cka1	Fission yeast	0	4.44E-10	0.9584
Q9Z0H0	Cdc7	Mouse	17.02	0	0.9829	P19454	CKA2	Baker’s yeast	1.179	1.18E-10	0.9522
P50582	hsk1	Fission yeast	14.59	3.32E-24	0.9818	P15790	CKA1	Baker’s yeast	0	2.28E-08	0.9444
Q54DK3	cdc7	Slime mold	70.02	3.81E-28	0.9295	O23236	MPK14	Mouse-ear cress	1.385	7.45E-09	0.9360
Q9CAI5	CKL2	Mouse-ear cress	27.09	1.68E-12	0.7495	P20427	CSNK2A2	Bovine	0.2857	1.64E-07	0.9344
P43565	RIM15	Baker’s yeast	39.77	6.42E-12	0.7046	P19784	CSNK2A2	Human	0.2857	1.67E-07	0.9343
Q54P47	ndrC	Slime mold	52.50	5.16E-12	0.6973	Q39027	MPK7	Mouse-ear cress	0.8152	8.86E-08	0.9320
Q8SRU0	CKA1	Microsporidian parasite	0	5.22E-11	0.6890	O54833	Csnk2a2	Mouse	0.8571	1.61E-07	0.9293
Q6Z8C8	CDKF-4	Rice	35.51	1.74E-11	0.6833	Q08467	CKA1	Mouse-ear cress	0.9779	1.67E-07	0.9281
Q9ZUP4	CKL5	Mouse-ear cress	31.87	2.97E-11	0.6726	Q9AR27	HD6	Rice	1.201	1.37E-07	0.9268
P42158	CKL1	Mouse-ear cress	31.77	3.07E-11	0.6718	P28523	ACK2	Maize	1.204	1.39E-07	0.9268
Q5A3P6	PKH2	Yeast	52.05	1.62E-11	0.6686	P08181	CkIIalpha	Fruit fly	1.488	1.86E-07	0.9231
P19454	CKA2	Baker’s yeast	1.179	1.18E-10	0.6645	P06243	CDC7	Baker’s yeast	8.678	1.10E-29	0.9218
Q1ZXI5	DDB_G0278845	Slime mold	71.55	2.14E-11	0.6417	Q39021	MPK1	Mouse-ear cress	2.432	4.32E-08	0.9201
Q03407	PKH1	Baker’s yeast	34.33	9.40E-11	0.6380	P21869	N/A	Chicken	3.142	5.64E-08	0.9127
P38938	cek1	Fission yeast	19.20	1.59E-10	0.6379	Q40517	NTF3	Common tobacco	4.838709677	1.39E-07	0.894111822

### Occurrence of structured homologues of human proteins in other species

**Fig. 4:**
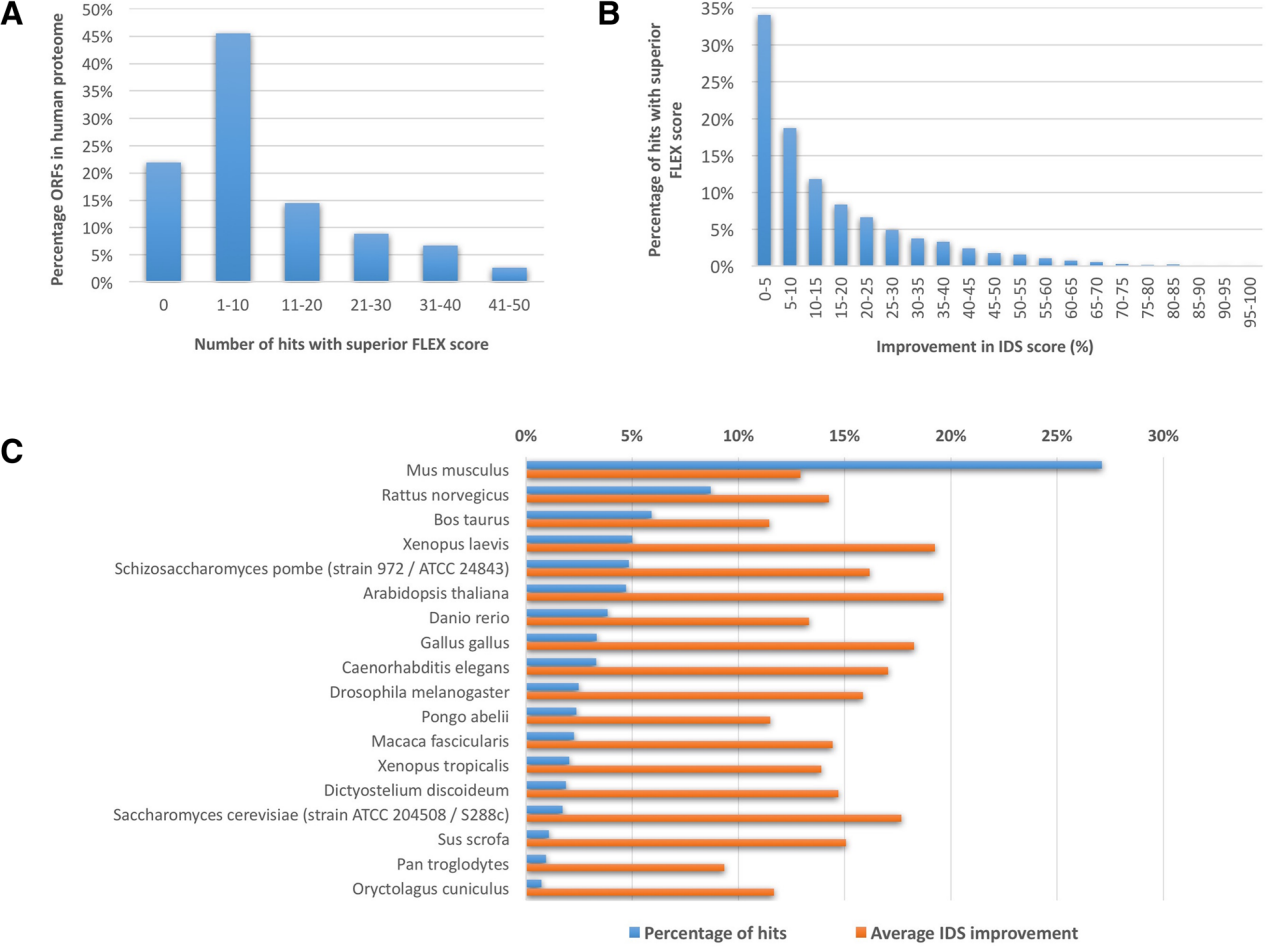
Results of a high-throughput BASILIScan search with every available ORF in the human proteome. The success of this search can be assessed by the number of homologous sequences found with a FLEX score higher than that of the query sequence (**a**). Limiting the results dataset to the queries which yielded at least one such homologue, the extent of improvement in IDS score can be plotted in 5% increments (**b**). Information about the most common species of the highest-scoring homologue for each query was also plotted (blue), along with the corresponding average difference in IDS (orange) (**c**). For full raw data, see Additional file 3

In order to learn about the scope of BASILIScan applicability in finding structured homologues of disorder-enriched proteins, every sequence of the human proteome (all 20,243 sequences available in UniprotKB/Swissprot) was subjected to an individual BASILIScan search with the same parameters as used for hCDC7 above. All human sequences were removed from the results database. This high-throughput search resulted in disorder prediction and scoring of nearly 100,000 protein sequences, and showed that a surprisingly large extent of the human proteome has homologues of superior intrinsic stability in other species. BASILIScan identified at least one homologue exhibiting a FLEX score higher than that of the query sequence for the vast majority of the human proteome (14,865 sequences or 78%) and ten or more such homologues for over 30% (Fig. 4a).

Importantly, in just under half of hits scored as promising – possessing FLEX scores higher than the query – the improvement of intrinsic disorder represented by the difference in IDS scores was very significant. The IDS score improvement of 10% or more was found in 47% of such BASILIScan-identified hits, while 27% of hits showed at least 20% improvement (Fig. 4b). The top hits resulting from the BASILIScan search belonged to 631 unique species, but by far the highest occurrence was displayed by mouse homologues (Fig. 4c). The improvement in IDS score appeared not to correlate with the hit frequency of occurrence in a given species.

## Discussion

As structural biology is evolving from tackling thermodynamically-stable, well-folded protein domains into much larger, multimeric and multicomponent protein systems, the need for rapid assessment and minimisation of intrinsic disorder becomes increasingly important. Software developed to assist recombinant expression construct design for predicting the regions of disorder (such as GlobPlot [9], or IUPRED [11]), maximising protein stability and crystallisibility through computing of parameters sourced from previously-solved crystal structures (XtalPred, [27]) and surface entropy reduction (SERp, [28]) have been used extensively. However, to the author’s knowledge, user-friendly and widely-accessible tools for high-throughput comparative analysis of intrinsic disorder patterns in related proteins have not been developed and explored for the purpose of recombinant expression construct design. This is tackled by the novel homologue search and scoring system offered by BASILIScan, with the disorder predictions based on the widely-adopted IUPRED algorithm. The key advantage of BASILIScan over the pre-exisiting software is the parallel analysis of disorder predictions for multiple related proteins at a time: both globally (the “trace” function) and residue-by-residue, as a heatmap (the “align” function). When the aim is to identify regions of disorder, such alignments can provide information on the conservation of the putatively-disordered region – and hence, how likely it is that the removal of such a fragment will not affect the structure or function of the protein.

## Conclusions

The functionalities implemented in BASILIScan serve two main experimental purposes: identification of homologues with diminished intrinsic disorder; and recognition of disordered regions suitable for deletion due to limited conservation. For both applications, the accessible user interface and the adjustable parametrisation are invaluable for identification of the most promising candidates and regions. Furthermore, as demonstrated by the high-throughput BASILIScan screen of the human proteome, identification of a more intrinsically-stable homologue of an IDP is likely to be feasible in most cases. Therefore, BASILIScan should be a valuable and easily-accessible resource for more streamlined rational expression construct design approaches and study of IDPs.

## Additional files

Additional file 1: Supplementary tables and figures. (DOCX 270 kb)

Additional file 2: BASILIScan search with human CDC7 kinase (Uniprot ID: O00311) against all vertebrate sequences available from UniprotKB (both Swissprot and TrEMBL). (CSV 263 kb)

Additional file 3: Data used to generate Fig. 4. Summary of the BASILIScan search with all human protein sequences available from UniprotKB/Swissprot against the UniprotKB/Swissprot repository. (CSV 1418 kb)

## Abbreviations

BLAST: Basic local alignment search tool
CDC7: Cell division cycle protein kinase 7
GUI: Graphical user interface
IDP: Intrinsically-disordered protein
IDS: Intrinsic disorder score
NMR: Nuclear magnetic resonance

## References

[1] Uversky VN (2016). Dancing protein clouds: The strange biology and chaotic physics of intrinsically disordered proteins. J Biol Chem.

[2] Pentony MM, Jones DT (2010). Modularity of intrinsic disorder in the human proteome. Proteins.

[3] Tokmakov AA (2015). Content of intrinsic disorder influences the outcome of cell-free protein synthesis. Sci Rep.

[4] Paz A (2008). Biophysical characterization of the unstructured cytoplasmic domain of the human neuronal adhesion protein neuroligin 3. Biophys J.

[5] Hinds MG (2007). Bim, Bad and Bmf: intrinsically unstructured BH3-only proteins that undergo a localized conformational change upon binding to prosurvival Bcl-2 targets. Cell Death Differ.

[6] Uversky VN (2013). A decade and a half of protein intrinsic disorder: biology still waits for physics. Protein Sci.

[7] Li X, Romero P, Rani M, Dunker AK, Obradovic Z (1999). Predicting protein disorder for N-, C-,and Internal Regions. Genome Inform Ser Workshop Genome Inform.

[8] Prilusky J (2005). FoldIndex: a simple tool to predict whether a given protein sequence is intrinsically unfolded. Bioinformatics.

[9] Linding R, Russell RB, Neduva V, Gibson TJ (2003). GlobPlot: exploring protein sequences for globularity and disorder. Nucleic Acids Res.

[10] Jones DT, Ward JJ (2003). Prediction of disordered regions in proteins from position specific score matrices. Proteins.

[11] Dosztanyi Z, Csizmok V, Tompa P, Simon I (2005). IUPred: web server for the prediction of intrinsically unstructured regions of proteins based on estimated energy content. Bioinformatics.

[12] Haynes C (2006). Intrinsic disorder is a common feature of hub proteins from four eukaryotic interactomes. PLoS Comput Biol.

[13] Habchi J, Longhi S (2015). Structural disorder within Paramyxoviral nucleoproteins and phosphoproteins in their free and bound forms: from predictions to experimental assessment. Int J Mol Sci.

[14] Cho Y, Gorina S, Jeffrey PD, Pavletich NP (1994). Crystal structure of a p53 tumor suppressor-DNA complex: understanding tumorigenic mutations. Science.

[15] Barski M, et al. Rift Valley fever phlebovirus NSs protein core domain structure suggests molecular basis for nuclear filaments. Elife. 2017;6. 10.7554/eLife.29236 PMC5601994 28915104

[16] Nagi AD, Regan L (1997). An inverse correlation between loop length and stability in a four-helix-bundle protein. Fold Des.

[17] Cock PJ (2009). Biopython: freely available Python tools for computational molecular biology and bioinformatics. Bioinformatics.

[18] Altschul SF, Gish W, Miller W, Myers EW, Lipman DJ (1990). Basic local alignment search tool. J Mol Biol.

[19] The UniProt C (2017). UniProt: the universal protein knowledgebase. Nucleic Acids Res.

[20] Pushker R, Mooney C, Davey NE, Jacque JM, Shields DC (2013). Marked variability in the extent of protein disorder within and between viral families. PLoS One.

[21] Pajkos M, Meszaros B, Simon I, Dosztanyi Z (2012). Is there a biological cost of protein disorder? Analysis of cancer-associated mutations. Mol BioSyst.

[22] Meszaros B, Dosztanyi Z, Simon I (2012). Disordered binding regions and linear motifs--bridging the gap between two models of molecular recognition. PLoS One.

[23] Edwards RJ, Davey NE, Shields DC (2007). SLiMFinder: a probabilistic method for identifying over-represented, convergently evolved, short linear motifs in proteins. PLoS One.

[24] Thompson JD, Higgins DG, Gibson TJ (1994). CLUSTAL W: improving the sensitivity of progressive multiple sequence alignment through sequence weighting, position-specific gap penalties and weight matrix choice. Nucleic Acids Res.

[25] Labib K (2010). How do Cdc7 and cyclin-dependent kinases trigger the initiation of chromosome replication in eukaryotic cells?. Genes Dev.

[26] Hughes S (2012). Crystal structure of human CDC7 kinase in complex with its activator DBF4. Nat Struct Mol Biol.

[27] Slabinski L (2007). XtalPred: a web server for prediction of protein crystallizability. Bioinformatics.

[28] Goldschmidt L, Cooper DR, Derewenda ZS, Eisenberg D (2007). Toward rational protein crystallization: a web server for the design of crystallizable protein variants. Protein Sci.

