# Supplementary material for: BASILIScan: a tool for high-throughput analysis of intrinsic disorder patterns in homologous proteins
Source: BMC Genomics. 2018 Dec 11;19:902. doi: 10.1186/s12864-018-5322-5 (PMC6290515; doi:10.1186/s12864-018-5322-5)
Supplement: Supplementary file 1 — Supplementary tables and figures. (DOCX 270 kb) [file 12864_2018_5322_MOESM1_ESM.docx]

**Supplementary tables**

| **Identifier** | **GeneID** | **IDS** | **E-value** | **Identity** | **Similarity** | **FLEX_score** |
| --- | --- | --- | --- | --- | --- | --- |
| **O00311** | CDC7 | 13.763 | 0 | 1.000 | 1.000 | 0.931 |
| **Q9Z0H0** | Cdc7 | 17.021 | 0 | 0.809 | 0.874 | 0.915 |
| **P06243** | CDC7 | 8.679 | 1.10E-29 | 0.134 | 0.191 | 0.956 |
| **Q8SR83** | CDC7-2 | 3.704 | 1.54E-28 | 0.174 | 0.268 | 0.981 |
| **Q8SR85** | CDC7-1 | 3.704 | 1.71E-28 | 0.174 | 0.268 | 0.981 |
| **Q54DK3** | cdc7 | 70.028 | 3.80E-28 | 0.057 | 0.083 | 0.650 |
| **P50582** | hsk1 | 14.596 | 3.31E-24 | 0.128 | 0.195 | 0.925 |
| **Q9UQY9** | spo4 | 2.564 | 1.57E-21 | 0.147 | 0.235 | 0.981 |
| **Q9CAI5** | CKL2 | 27.097 | 1.67E-12 | 0.110 | 0.176 | 0.740 |
| **Q54P47** | ndrC | 52.509 | 5.14E-12 | 0.050 | 0.079 | 0.599 |
| **P43565** | RIM15 | 39.774 | 6.40E-12 | 0.050 | 0.089 | 0.659 |
| **Q5A3P6** | PKH2 | 52.059 | 1.61E-11 | 0.052 | 0.102 | 0.585 |
| **Q6Z8C8** | CDKF-4 | 35.512 | 1.73E-11 | 0.105 | 0.174 | 0.666 |
| **Q1ZXI5** | DDB_G0278845 | 71.556 | 2.13E-11 | 0.037 | 0.057 | 0.483 |
| **Q9ZUP4** | CKL5 | 31.871 | 2.97E-11 | 0.113 | 0.189 | 0.677 |
| **P42158** | CKL1 | 31.778 | 3.06E-11 | 0.109 | 0.178 | 0.676 |
| **Q8SRU0** | CKA1 | 0.000 | 5.20E-11 | 0.125 | 0.226 | 0.827 |
| **Q03407** | PKH1 | 34.334 | 9.37E-11 | 0.059 | 0.097 | 0.646 |

**Table S1.** Summary of BASILIScan results for homologues of human CDC7 kinase (Uniprot/Swissprot identifier O00311). Search parameters and the scoring system are described in “Results”. Calculations of the IDS and FLEX score parameters are described in the “Design and Implementation” section.


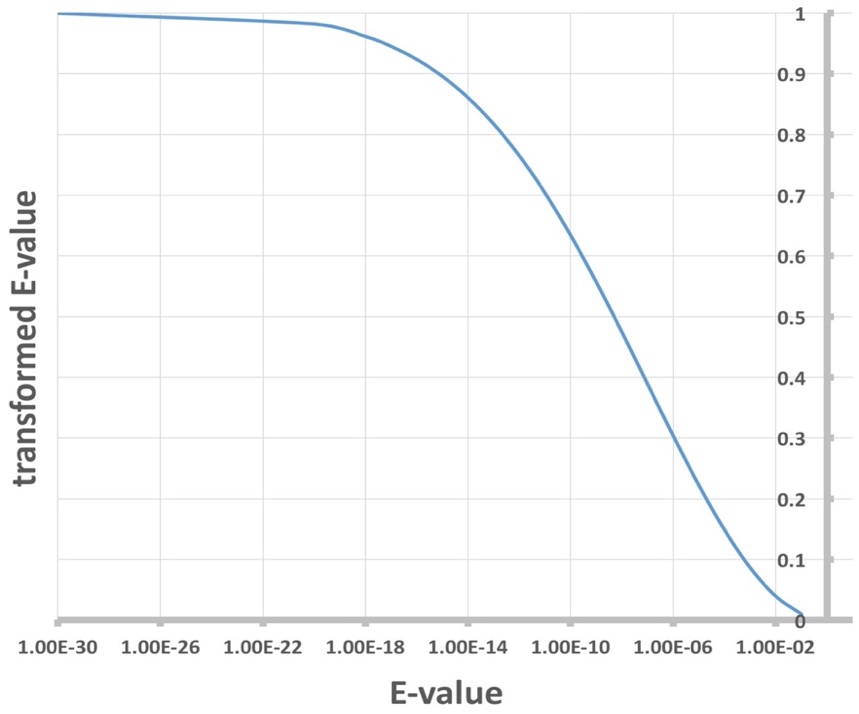
**Supplementary figures**


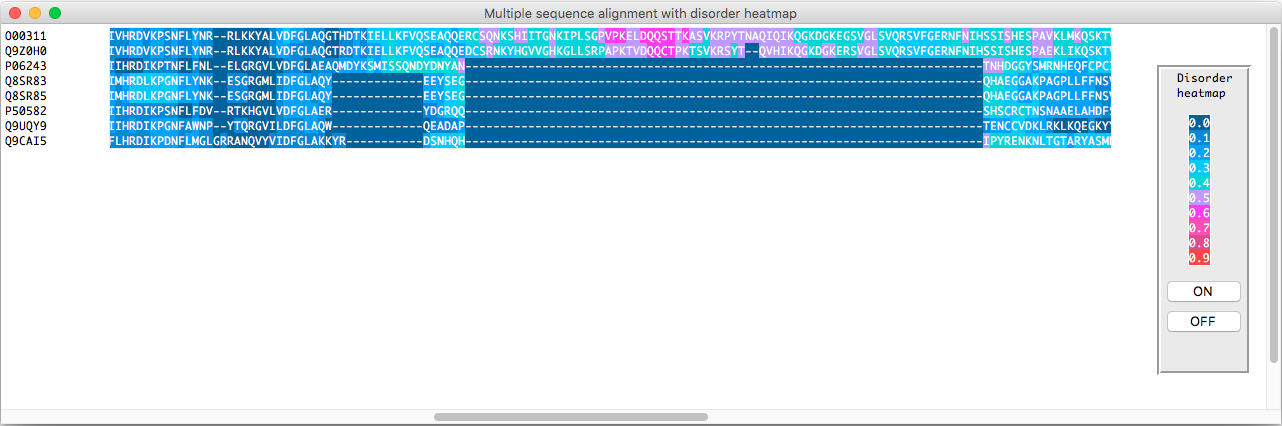
**Supplementary figure S1.** For more accurate scoring purposes, a hyperbolic transform is applied to the BLAST-derived E parameter, resulting in a value bound from 0 to 1 for E-values of below 1. The function is near-linear for E-values of between 10^-14^ and 10^-3^, while both far ends reach a plateau. The transformed E-value is then used for calculation of the FLEX score.

**Supplementary figure S2.** Eight highest-scoring hits from a BASILIScan search against hCDC7 kinase (Uniprot/Swissprot identifier O00311) aligned with the “Align” module and with the calculated intrinsic disorder overlayed on the alignment as a heatmap. In this case, the absence of an extended, highly-disordered fragment in the bottom five sequences is apparent.
